# Mycobacterial RNA isolation optimized for non-coding RNA: high fidelity isolation of 5S rRNA from *Mycobacterium bovis* BCG reveals novel post-transcriptional processing and a complete spectrum of modified ribonucleosides

**DOI:** 10.1093/nar/gku1317

**Published:** 2014-12-24

**Authors:** Fabian Hia, Yok Hian Chionh, Yan Ling Joy Pang, Michael S. DeMott, Megan E. McBee, Peter C. Dedon

**Affiliations:** 1 Singapore MIT Alliance for Research and Technology, 1 CREATE Way, 138602, Singapore; 2 Department of Microbiology and Immunology Programme, National University of Singapore, 117456, Singapore; 3 Department of Biological Engineering, Massachusetts Institute of Technology, Cambridge, MA 02139, USA; 4 Center for Environmental Health Sciences, Massachusetts Institute of Technology, Cambridge, MA 02139, USA

## Abstract

A major challenge in the study of mycobacterial RNA biology is the lack of a comprehensive RNA isolation method that overcomes the unusual cell wall to faithfully yield the full spectrum of non-coding RNA (ncRNA) species. Here, we describe a simple and robust procedure optimized for the isolation of total ncRNA, including 5S, 16S and 23S ribosomal RNA (rRNA) and tRNA, from mycobacteria, using *Mycobacterium bovis* BCG to illustrate the method. Based on a combination of mechanical disruption and liquid and solid-phase technologies, the method produces all major species of ncRNA in high yield and with high integrity, enabling direct chemical and sequence analysis of the ncRNA species. The reproducibility of the method with BCG was evident in bioanalyzer electrophoretic analysis of isolated RNA, which revealed quantitatively significant differences in the ncRNA profiles of exponentially growing and non-replicating hypoxic bacilli. The method also overcame an historical inconsistency in 5S rRNA isolation, with direct sequencing revealing a novel post-transcriptional processing of 5S rRNA to its functional form and with chemical analysis revealing seven post-transcriptional ribonucleoside modifications in the 5S rRNA. This optimized RNA isolation procedure thus provides a means to more rigorously explore the biology of ncRNA species in mycobacteria.

## INTRODUCTION

Infection with *Mycobacterium tuberculosis* (Mtb) represents one of the most widespread microbial diseases, with nearly one-third of the world's population showing signs of exposure, more than 20 million people actively infected and almost 80% of the population of some countries testing positive in tuberculin tests (1,2). This rate of infection is due to both a paucity of diagnostic tools (3–6) and ineffective chemotherapy in the face of emerging drug-resistance (7,8), both of which reflect poor understanding of mycobacterial biology and host–pathogen interactions (9,10). One feature of mycobacterial biology that has hampered investigations is a thick, waxy cell wall consisting of a network of peptidoglycans, arabinogalactans, mycolic acids and polysaccharides (11,12), which makes mycobacteria resistant to lysis by most commercial chaotropic or cell lysis reagents and poses challenges to the rigorous purification of cellular biomolecules. We are concerned here with the isolation of non-coding RNA (ncRNA). The importance of rigorous ncRNA purification is illustrated by recent advances in RNA sequencing and bioinformatics that have led to the discovery of disease-relevant ncRNA species in mycobacteria (13,14), while critical features of modified ribonucleosides in transfer RNA (tRNA) and ribosomal RNA (rRNA) are known to play a role in adaptive responses to stress (15–18). In all cases, the systems-level analysis of ncRNA requires unbiased isolation of RNA with sequence integrity and relative quantity intact. Numerous methods for mycobacterial RNA isolation have been developed that include liquid or solid-phase extraction following cell lysis by either sonication, enzymatic hydrolysis, chemical treatment, French pressure cell rupture or bead-beating (19–22). However, there has been no rigorous optimization of mycobacterial RNA isolation techniques to ensure purification of the full spectrum of ncRNA species with quantitative and qualitative fidelity. Furthermore, in addition to recognized size- and sequence-dependent biases in the isolation of specific ncRNA species (23), these methods require large quantities of cells or have time-consuming steps that can lead to degradation or enzymatic modification of the RNA (24). To address these problems, we developed an optimized method for efficient isolation of all types of ncRNA from mycobacteria with high biological fidelity. Using *Mycobacterium bovis* Bacille Calmette-Guérin (BCG) as the model mycobacterial species, the method represents a combination of bead-beating with phenol-chloroform and solid-phase extraction steps optimized for both yield and quality of tRNA, 5S, 16S and 23S rRNA, as well as mRNA for seven genes. Application of the method to BCG revealed hypoxia-induced alterations of the relative quantities of 16S and 23S rRNA, a novel post-transcriptional processing of 5S rRNA, and the first complete analysis of the full set of modified ribonucleosides in mycobacterial 5S rRNA.

## MATERIALS AND METHODS

### Bacterial cultures

For exponentially growing mycobacteria, *M. bovis* BCG (str. Pasteur 1173P2; BCG) was grown at 37°C in a shaking incubator in Middlebrook 7H9 broth (Difco, BD Diagnostics, Sparks, MD, USA) to an OD_600_ of 0.6–0.8. BCG cells in a hypoxia-induced non-replicative state were obtained using the Wayne model adapted from Low *et al*. (25). Briefly, a culture of exponentially growing BCG in Dubos broth (Difco) was diluted to an OD_600_ of 0.05 and placed in a tightly sealed (latex-lined cap) 1-l glass flask (Duran, Mainz, Germany) with stirring at 170 rpm and air headspace volume of 450 ml. With a plateau in cell growth at 7 days (as determined by OD_600_), induction of a non-replicative state was confirmed by the absence of change in OD_600_ for the next 14 days, at which point the cells were harvested (Supplementary Figure S1).

### Development of the RNA isolation method

The optimized RNA isolation protocol was developed around a simple framework consisting of lysis by bead-beating in a denaturing extraction solvent, with subsequent RNA retrieval from the solvent. Using this framework, we systematically tested a variety of conditions and parameters, including extraction reagent, bead beating conditions and RNA retrieval method, to define an optimal protocol to extract all types of ncRNA from BCG. Two nucleic acid extraction reagents were considered: TRIzol (Invitrogen, Carlsbad, CA, USA) and phenol:chloroform:isoamyl alcohol (Qiagen, Hilden, Germany). While harshly denaturing, these reagents are insufficient to lyse mycobacterial cells. To assist in lysing cells, mechanical disruption by bead beating on a reciprocal shaker was chosen, with optimization of bead size, extraction solvent volumes and duration. For retrieval of RNA from the extraction solvent following mechanical disruption, we compared simple isopropanol precipitation of RNA to solid-phase isolation of RNA using the Purelink miRNA Isolation Kit (Invitrogen, Carlsbad, CA, USA). A summary of all conditions tested and their respective nucleic acid yields before DNase I treatment as assessed by UV spectroscopy is detailed in Supplementary Table S1; the analyses were performed in three biological replicates.

Supplementary Figure S2 illustrates the final optimized ncRNA isolation method, which was applied to BCG cultures growing exponentially or in a hypoxia-induced non-replicative state. Cells are harvested from culture medium by centrifugation at 4000 x g for 5 min at 4°C. Pellets containing 10^9^ cells were washed in phosphate-buffered saline (PBS) containing 0.5% Tween 80 to remove media debris that could interfere with RNA isolation and resuspended in 1 ml of ice-cold phenol:chloroform:isoamyl alcohol (volume ratio 25:24:1; saturated with 10-mM Tris, 1 mM EDTA, pH 8) and 400 μl of 2-M sodium acetate (pH 5.2). The suspension was combined with 100 μl of 0.1-mm zirconia-silica beads (Biospec, Bartlesville, OK, USA) in a 2-ml screw-capped tube and vigorously shaken on a Qiagen Tissuelyser II (Qiagen, Hilden, Germany) with chambers pre-chilled to −20°C to account for heating to ambient temperature by the end of the 30-min run at a frequency of 30 Hz. After centrifugation (12 000 x g, 15 min, 4°C), the aqueous phase (∼400 μl) was transferred into a new tube to which 215 μl of 100% ethanol was added with mixing and the solution loaded onto a Purelink miRNA Isolation Kit (Invitrogen, Carlsbad, CA, USA) column (column #1) to retain large RNA species. Following centrifugation of the column (12 000 x g, 1 min), the flow-through containing small RNAs was collected, placed on ice, combined with 700 μl of 100% ethanol and loaded onto a second Purelink miRNA Isolation column (column #2) to retain the small RNA species. The column was again centrifuged (12 000 x g, 1 min) and the flow-through discarded. Both columns (#1 and #2) were washed twice with washing buffer provided with the kit. RNase-free water (50 μl) was added to the columns, which were incubated at ambient temperature for 2 min followed by centrifugation (12 000 x g, 1 min). The eluates from columns #1 and #2 contained mainly large and small RNA, respectively, and these were combined to recreate a total RNA preparation. Aliquots of each RNA sample were adjusted to a concentration of 1 ng/μl and thee integrity and relative quantities of the RNA species in the sample were assessed using an Agilent 2100 Bioanalyzer (Agilent Technologies, Santa Clara, CA, USA). Column eluates were stored at −80°C.

### HPLC purification of individual ncRNA species

Individual RNA species were purified by size-exclusion (SEC) high-performance liquid chromatography (HPLC), as described in a recent publication (26). Following addition of 30 units of DNAse I (Qiagen, Hilden, Germany) to each sample, the total RNA preparation in 20 μl was injected onto an Agilent Bio SEC3 column (300 Å, 7.8 × 100 mm; Agilent, Foster City, CA, USA) attached to an Agilent 1200 HPLC system to resolve tRNA and 5S rRNA species. For 16S and 23S rRNA, an Agilent Bio SEC5 column (1000 Å, 7.8 × 100 mm) was used. Both columns were eluted with an isocratic gradient with 100-mM ammonium acetate (pH 7.5) at 0.5 ml/min and 60°C with RNA elution monitored by UV absorbance. Peak resolution (R) was calculated using the following equation where tRA and tRB are the retention times for peaks A and B, respectively, and WA and WB are the peak base widths for peaks A and B, respectively:

\begin{equation*} R = 2\left( {\frac{{{\rm tRB} - {\rm tRA}}}{{{\rm WA} + {\rm WB}}}} \right). \end{equation*}

Fractions containing individual RNA species were analyzed on an Agilent 2100 Bioanalyzer. The 5S rRNA fraction was concentrated and desalted using a 2000-Da molecular weight cut-off spin filter (Sartorius-Stedim, Goettingen, Germany). Purified 5S rRNA was quantified by integrating the UV absorbance for the 5S rRNA peak in HPLC profiles and interpolating concentration from linear standard curves prepared with purified 28S rRNA (CCRF-SB cells), as shown in Supplementary Figure S3.

### Sequencing of BCG 5S rRNA

To confirm the identity of the purified 5S rRNA, the 3′ end of 25 pmol of the RNA was ligated to 50 pmol of a pre-adenylated DNA adaptor (IDT, Coralville, IA, USA) using 1.5 μg of RNA ligase 2 (truncated K227Q, NEB, Beverly, MA, USA) with incubation at 22°C for 16 h. The ligated product was purified by SEC3 HPLC and then reverse transcribed with 200 U of Superscript III RT (Invitrogen, Carlsbad, CA, USA). The resulting cDNA was ligated to 50 pmol of another pre-adenylated 3′ adaptor (IDT, Coralville, IA, USA) using 10 U of T4 RNA ligase 1 (NEB, Beverly, MA, USA) with incubation at 4°C for 16 h. Following SEC3 HPLC purification, the ligated cDNA was PCR amplified, TA cloned (Genewiz, South Plainfield, NJ, USA), and sequenced to confirm its identity (Genewiz, South Plainfield, NJ, USA). Sequence analysis was performed with Nucleotide BLAST (NCBI) against the annotated full genome sequence of the same BCG strain (NC_008769.1). The cDNA sequence from mature 5S rRNA is available on GenBank (Accession number KC203333).

### Analysis of mRNA by quantitative real-time polymerase chain reaction

To compare the mRNA yield from the optimized RNA isolation approach to that of the conventional TRIzol method, RNA was isolated from exponentially growing BCG cultures using TRIzol or the optimized RNA isolation method and DNA was removed using the TURBO DNA-free Kit (Ambion, Life Technologies, Carlsbad, CA, USA). RNA (50 ng/μl) was reverse transcribed using the iScript cDNA synthesis kit (Bio-Rad, Hercules, CA, USA) according to the manufacturer's instructions. The reverse transcription program was run as follows: 25°C for 5 min, 42°C for 30 min and 85°C for 5 min, followed by a cooling step at 4°C. Two-step real-time quantitative polymerase chain reaction (qPCR) was then performed using the SsoAdvanced universal SYBR Green supermix (Bio-Rad, Hercules, CA, USA). Primer sequences and melting temperatures can be found in Supplementary Table S3. The qPCR program was run as follows: 95°C for 30 s followed by 40 cycles of denaturation at 95°C for 15 s and annealing/extension at 60°C for 30 s. A melting curve analysis consisting of 0.5°C increments from 65 to 95°C was performed for all reactions to ascertain the specificity of the primers.

### Identification and characterization of modified ribonucleosides in BCG 5S rRNA

The full spectrum of modified ribonucleosides in the purified 5S rRNA was characterized by analysis of ribonucleoside hydrolysates of the RNA by chromatography-coupled quadrupole time-of-flight and triple quadrupole mass spectrometry, using methods described previously (15,26,27). Briefly, purified BCG 5S rRNA was enzymatically hydrolyzed in the presence of antioxidants and deaminase inhibitors and the ribonucleosides resolved by HPLC using a Hypersil GOLD aQ Analytical HPLC Column (100 × 2.1 mm, 1.9-μm particle size) (Thermo Scientific, Wilmington, DE, USA) coupled to either an Agilent 6460 triple quadrupole mass spectrometer or an Agilent 6520 quadrupole time-of-flight mass spectrometer (LC-MS/MS) with ESI ionization operated in positive ion mode. The identity of individual ribonucleosides was confirmed by comparison with synthetic standards for HPLC retention time, exact molecular weight and collision-induced dissociation (CID) fragmentation patterns. In cases where a standard was not available, a putative structure was inferred from exact molecular weight and CID fragmentation patterns.

### Statistical analysis

Statistical analysis for comparison of qPCR *C*_T_ values for the RNA isolation methods and quantities of RNA species in exponentially growing and hypoxic, non-replicative (Wayne day 21) cultures was performed using Student's *t*-test with significance set at *P* ≤ 0.05.

## RESULTS

Given the challenges of working with mycobacteria, we sought to develop a method for purification of ncRNA that optimized the cell lysis, RNA extraction and RNA purification steps to yield the full spectrum of intact ncRNA species in quantities that reflect their abundances *in vivo*. The workflow shown in Supplementary Figure S2 represents the culmination of a series of optimization studies in which different procedures were assessed for the yield of RNA, the relative quantities of the various ncRNA species and the quality of the RNA in terms of degradation. Subsequent analysis of the isolated ncRNA species from BCG revealed several novel features of RNA biology in mycobacteria.

### Optimization of RNA isolation parameters

With all of the optimization data shown in Supplementary Table S1, optimization of the ncRNA isolation method was initiated with a comparison of two standard extraction reagents, TRIzol and the 25:25:1 mixture of phenol, chloroform and isoamyl alcohol. An extraction reagent was added to the cells prior to bead beating to quench adventitious enzymatic reactions during cell lysis, including nucleases and RNA modification enzymes. Given the evidence for biased isolation of miRNA with TRIzol (23), we compared the yields of the major ncRNA species in BCG with TRIzol and phenol:chloroform:isoamyl alcohol. A 77% increase in nucleic acid recovery was obtained with phenol:chloroform:isoamyl alcohol compared to TRIzol (Supplementary Table S1). Since phenol:chloroform:isoamyl alcohol saturated with 10-mM Tris, pH 8.0, also extracts DNA, it is possible that part of the observed increase in nucleic acid mass results from DNA contamination. However, the yield of longer RNA species, in particular 16S and 23S rRNA, was significantly reduced using traditional acid phenol or TRIzol-based RNA extraction methods (Supplementary Figure S4). Hence, it was preferable to use the phenol:chloroform:isoamyl alcohol approach to obtain all cellular RNA species in high yield and in proportions that truly reflect their abundances *in vivo*, with contaminating DNA subsequently removed by DNAse I digestion or HPLC purification, without sacrificing longer RNA species (Supplementary Figure S5).

Next, we optimized for cell lysis, comparing the RNA yield obtained using 0.5- and 1.0-mm glass beads and 0.1-mm zirconia-silica beads. While 0.5-mm glass beads offered a 61% increase in nucleic acid yield against 1.0-mm glass beads, we observed an additional 37% increase in RNA yield with 0.1-mm zirconia-silica beads (Supplementary Table S1). Using 0.1-mm zirconia-silica beads, we optimized for the duration of bead beating. A duration of 10 min was sufficient for lysis of mycobacterial cells using the TissueLyser II, while a duration of 30 min was required for the TissueLyser LT. Use of a French press was ruled out due to the time required for the lysis process (>15 min) in the absence of denaturants, which allows enzymatic reactions to affect the spectrum of tRNA and rRNA modifications or cause RNA degradation.

Finally, to recover RNA from the extraction solvents, we chose the Purelink miRNA Isolation Kit system over isopropanol-based RNA precipitation. While the Purelink columns were optimized for use with TRIzol, we modified the manufacturer's protocol to use phenol:chloroform:isoamyl alcohol instead, which allowed for the maximal recovery of both large and small RNA. Using 35% ethanol, larger RNA species were recovered on column #1 while smaller RNA species were recovered on column #2 using 70% ethanol.

### Qualitative assessment of the optimized ncRNA isolation method

**Figure 1.**
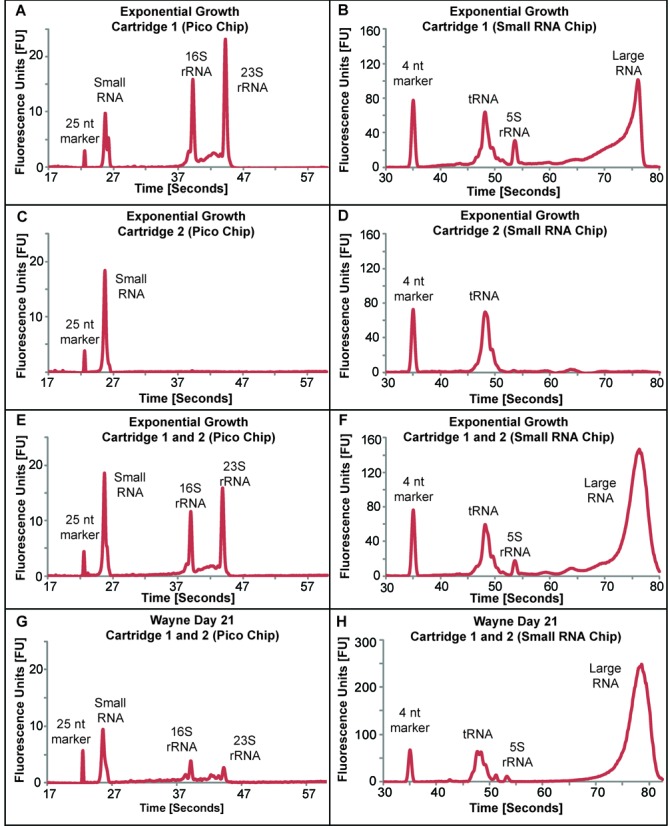
Representative Bioanalyzer electropherograms for BCG RNA recovered from Purelink miRNA Isolation columns #1 and #2. Electropherograms from column #1 RNA resolved on (**A**) a Pico Chip and (**B**) a Small RNA Chip, and from column #2 RNA resolved on (**C**) a Pico Chip and (**D**) a Small RNA Chip. Electropherograms for the combined RNA from columns #1 and #2 resolved on (**E**) a Pico Chip and (**F**) a Small RNA Chip. RNA isolated from BCG subjected to hypoxic stress (combined columns #1 and #2) was resolved on (**G**) a Pico Chip and (**H**) a Small RNA Chip. The electropherogram profiles presented here are representative of three independent experiments. Note that the small RNA chip on the Agilent Bioanalyzer 2100 system is not optimized to read large RNA species (>150 nucleotides). Thus the amount of RNA in the ‘large RNA’ peak cannot be accurately quantified.

To assess the performance of the optimized RNA isolation method and the quality of the isolated ncRNA, the method was applied to BCG cells growing exponentially and to cells arrested in a hypoxia-induced non-replicative state that mimics the granuloma of pulmonary mycobacterial infections. The quality and relative quantities of the various ncRNA species in total RNA extracted from BCG were assessed using a Bioanalyzer with either the Agilent Pico Chip for large RNA (16S, 23S) and the Agilent Small RNA Chip for small RNA species (<200 nt; miRNA-sized, tRNA, 5S rRNA). As shown in Figure 1, the peaks corresponding to individual ncRNA species were sharp and distinguishable. From the exponentially growing cultures, the eluate from column #1 contained 23S rRNA, 16S rRNA, 5S rRNA and tRNA (Figure 1A and B), whereas column #2 eluate contained predominantly tRNA (Figure 1C and D). As a numeric index of the quality of the isolated RNA, the RNA integrity number (RIN) determined from the Bioanalyzer profile ranges from 10 for theoretically intact RNA to 1 for completely degraded RNA (28). For exponentially growing BCG, the RIN for RNA eluted from column #1 in four independent extractions was 9.0 ± 0.5, while the RIN for the combined eluates from columns #1 and #2 from five independent extractions was 8.5 ± 0.3. Interestingly, Bioanalyzer RNA profiles of the hypoxic cultures displayed lower relative intensities of the 16S and 23S rRNA peaks compared to those from exponentially growing cultures (Figure 1G), with comparable quantities of tRNA under both conditions (Figure 1H). The RIN for the combined eluates from columns #1 and #2 from four independent RNA extractions of hypoxic bacilli was 5.9 ± 0.2, which suggested a degree of degradation of both 23S and 16S rRNA peaks (Figure 1G).

To assess the performance of the Purelink miRNA Isolation columns, the proportions of ncRNA species in column eluates were calculated by manual integration of electropherogram peaks with Bioanalyzer 2100 Expert software. A comparison of the TRIzol and phenol:chloroform:isoamyl alcohol methods is summarized in Supplementary Table S2. Using this approach, large ncRNAs (16S and 23S, identified by size) were almost entirely recovered from column #1 along with a significant portion of the small ncRNAs (<200 nt), while small ncRNAs represented >95% of the RNA eluted from column #2. The small ncRNAs, whose identities were assigned based on size, consisted of three major species: miRNA-like species, tRNA and 5S rRNA. This retention of small ncRNAs on column #1 points to potential bias in the small ncRNAs recovered from column #2 as ‘small RNA’. However, by combining the eluates from both columns, the yield of all ncRNA species was maximized (Figure 1E and F), with small RNA, and mainly tRNA, as the dominant species of ncRNA in BCG (Supplementary Table S2A). For TRIzol however, small RNA was the predominant species of ncRNA, with tRNA consisting of more than 80% of the small RNA species (Supplementary Table S2B).

Considering both the RIN and the recovery of all ncRNAs from the columns using the phenol:chloroform:isoamyl alcohol approach, the results with exponentially growing BCG RNA suggest that the degradation apparent in hypoxic, non-replicative BCG is not the result of the RNA isolation procedure and instead reflects physiological processing of the RNA. These differences were next assessed more quantitatively.

### Application of the mycobacterial RNA isolation method in isolating mRNA

While optimized for the isolation of ncRNA, we investigated the possibility of utilizing our approach for mRNA isolation. Here, we performed qPCR against seven gene transcripts on samples derived from RNA isolation with our approach and samples derived from the conventional TRIzol RNA isolation approach. Analysis revealed that there were no significant differences in *C*_T_ values against five gene transcripts as illustrated in Table 1. The remaining two transcripts showed that our RNA isolation method yielded a lower *C*_T_ value (i.e. higher concentration of mRNA) compared to the TRIzol approach.

**Table 1.** *C*_T_ values obtained from qPCR analysis of samples derived from TRIzol and the optimized RNA isolation approach

	Average *C*_T_ value		
Gene	P:C:IAA (optimized approach)	TRIzol	*P*-value^a^
*hspX*	21.38 ± 0.20	23.60 ± 0.91	0.014
*ethA*	27.34 ± 0.11	27.74 ± 1.13	0.608
*fdxA*	20.62 ± 0.31	20.45 ± 0.56	0.663
*relA*	23.82 ± 0.33	25.08 ± 0.89	0.081
*sigA*	24.92 ± 0.15	24.70 ± 0.52	0.093
*dosR*	23.73 ± 0.36	25.13 ± 0.82	0.053
*dosS*	24.92 ± 0.32	26.53 ± 0.76	0.028

^a^An unpaired Student's *t*-test was performed for the samples derived from the two approaches. Data represent mean ± SD for three biological replicates.

### Application of the mycobacterial RNA isolation method: quantitative comparison of ncRNA species in non-replicative and exponentially growing BCG

**Figure 2.**
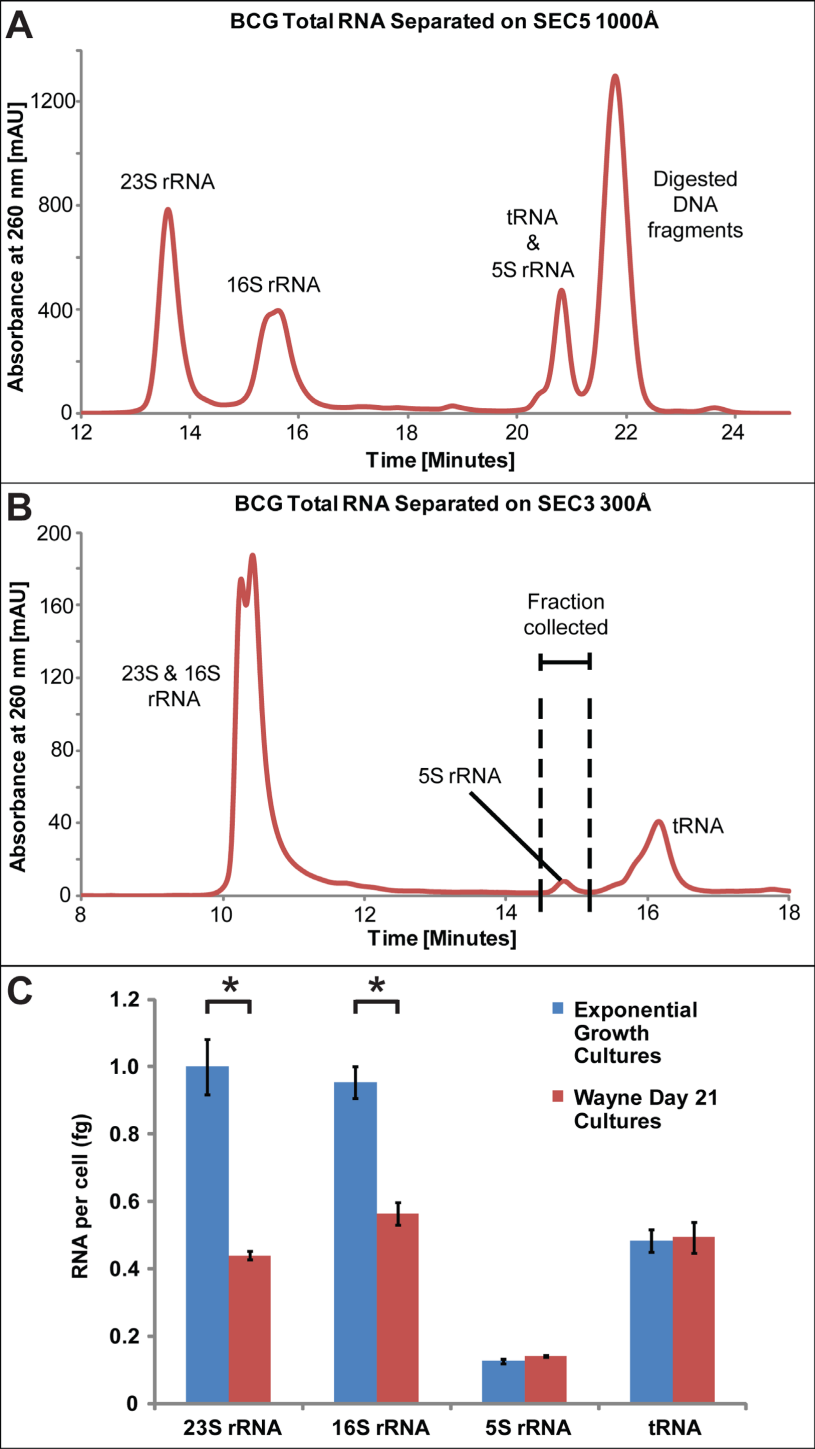
Size-exclusion HPLC chromatograms for individual RNA species. BCG total RNA was resolved on an Agilent Bio Size Exclusion Column SEC5, 1000 Å (**A**), or on an Agilent Bio Size Exclusion Column SEC3, 300 Å (**B**). Panels (A) and (B) are representative of six independent HPLC runs. (**C**) Cellular quantities of 5S, 16S and 23S rRNA and tRNA species based on HPLC UV absorbance interpolated from a standard curve based on purified human 28S rRNa. An asterisk indicates a significant difference in the quantity of RNA from exponentially growing cells compared to hypoxic cells on day 21 (unpaired T-test, *P* ≤ 0.05). Data represent mean ± SD for three biological replicates.

Given the differences in the relative quantities of 23S and 16S rRNAs between exponential and non-replicative cultures noted in the Bioanalyzer electropherograms (Figure 1E and G), we undertook a more rigorous quantitative analysis by HPLC resolution of total RNA extracts from BCG during large-scale purification of ncRNA species. As shown in Figure 2A and B, we were able to achieve baseline resolution (*R* = 3.9 ± 0.02) of 16S and 23S rRNAs using an Agilent size-exclusion HPLC column (SEC5 1000 Å), while tRNA and 5S rRNA were well resolved (*R* = 4.1 ± 0.04) using a solid phase with a smaller pore size (SEC3 300Å). The absolute quantities of individual ncRNA species were calculated by interpolating the UV absorbance peak areas for HPLC-resolved ncRNA species with a standard curve prepared from purified 28S rRNA. These quantities were then combined with cell counts from colony forming assays for cultures used in the RNA isolation to calculate the quantity of each rRNA species on a per cell basis. As shown in Figure 2C, the quantities of 16S and 23S rRNA from exponentially growing BCG were significantly higher than in hypoxia-induced non-replicative BCG (*P* ≤ 0.05), yet there was no difference in the quantities of 5S rRNA and tRNA for the two growth conditions. These data for cellular quantities of ncRNA are consistent with theoretical estimates of mycobacterial molecular composition (29).

### Sequence of BCG 5S rRNA

**Figure 3.**
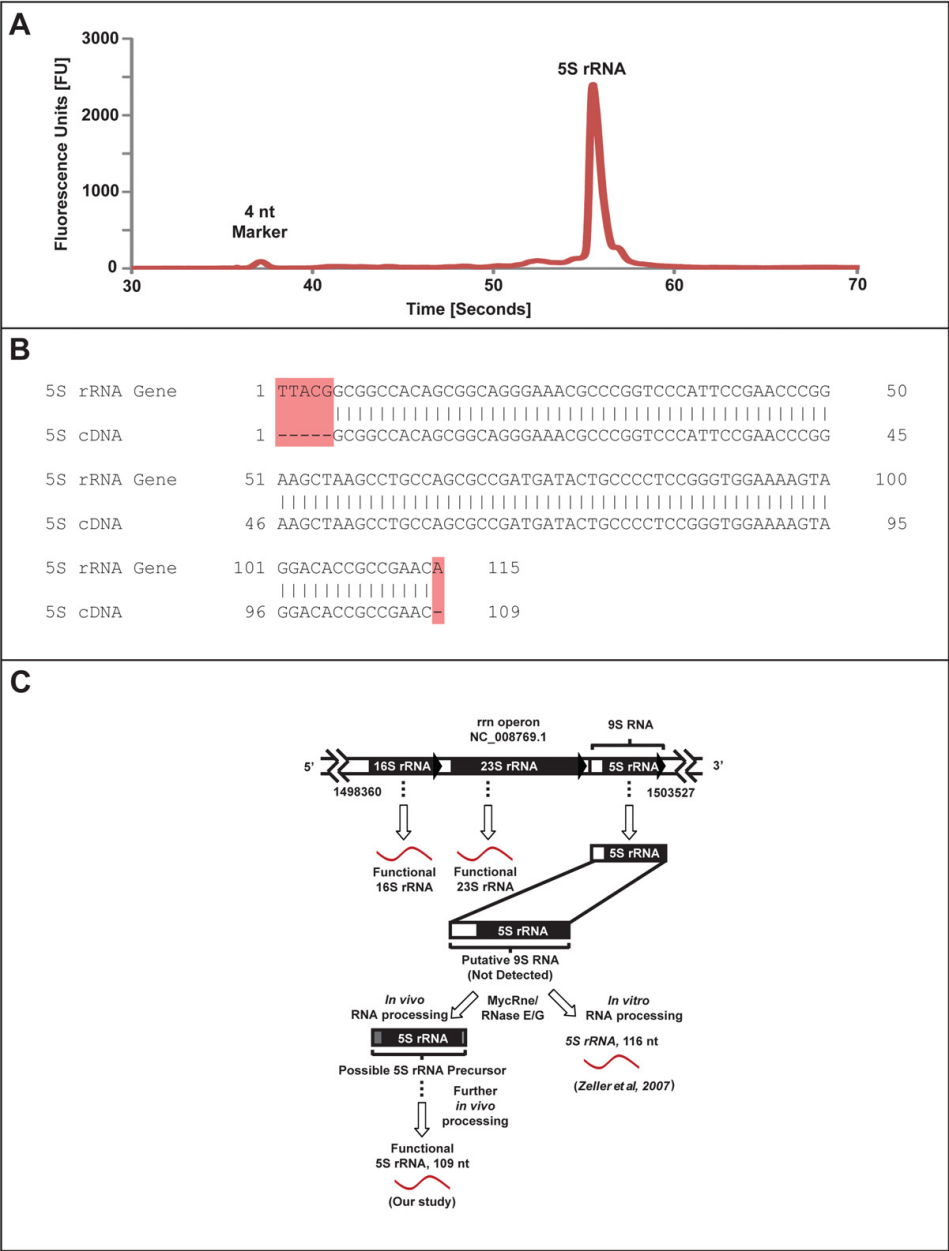
Purification and sequencing of BCG 5S rRNA and a proposed model for 5S rRNA processing. (**A**) A Bioanalyzer electropherogram for HPLC-purified 5S rRNA shows high purity. (**B**) Comparison of sequenced 5S rRNA against its annotated gene sequence. A total of six nucleotides are absent from the sequenced 5S rRNA (highlighted in red). (**C**) Possible fates of the putative 9S RNA, a precursor of functional 5S rRNA. Zeller *et al*. demonstrated *in vitro* processing of 9S rRNA to a 116-nt 5S rRNA by MycRNE/RNase E/G (44), while our analysis reveals different or further *in vivo* processing to yield a 109-nt functional 5S rRNA molecule.

Using the optimized RNA isolation method with BCG, we were able to reproducibly detect an RNA species with a size consistent with 5S rRNA. Though relatively abundant when compared to other bacterial small RNAs, the observation of 5S rRNA is notable in light of the fact that this species has never been isolated for direct sequencing. In other studies of mycobacterial ncRNA, small RNAs were identified by genomic analysis, by microarray detection of non-coding transcripts, by shotgun cloning of small RNAs or by co-purification with RNA-binding proteins (30–34). Here, pure 5S rRNA-containing fractions were collected from the HPLC resolution and the 5S rRNA subjected to sequencing to confirm its identity and to define the sequence of its mature, functional form. Analysis revealed a sequence of 109 nt that, in a Blast search, yielded an expect value (*E*) of 4 x *e*^−49^ and a maximal identity of 100% for several *Mycobacterium* species, including BCG, *M. tuberculosis, Mycobacterium canettii*, and *Mycobacterium africanum*. Comparison of the annotated BCG 5S rRNA sequence (*M. bovis BCG str. Pasteur 1173P2*, Gene ID: 4698678) and the cDNA sequence of our purified 5S rRNA revealed several differences in the 3′- and 5′-ends, as shown in Figure 3B. The purified 5S rRNA lacked 6 nt: positions 1 to 5 and position 115 in the annotated 5S rRNA sequence. These results suggest discrete processing of 5S rRNA from its genomic transcript as a 9S precursor to the functional sequence by a mycobacterial enzyme such as RNase E/G (MycRne), as illustrated in Figure 3C.

### The spectrum of modified ribonucleosides in BCG 5S rRNA

To take advantage of the ability to isolate 5S rRNA using the optimized method, we further explored 5S rRNA maturation by analyzing post-transcriptional modifications. Here, we used a highly sensitive chromatography-coupled mass spectrometric platform to identify modified ribonucleosides in RNA (15,26,27). As shown in Table 2, application of this platform to BCG 5S rRNA revealed seven modified ribonucleosides: N^1^-methyladenosine (m^1^A), N^6^,N^6^-dimethyladenosine (m^6,6^A), N^7^-methylguanosine (m^7^G), N^1^-methylguaonsine (m^1^G), 2′-*O*-methylguanosine (Gm) and inosine (I), each with distinct mass-to-charge transition ratios and retention times as shown in Supplementary Figure S6. The structures of six of the modified ribonucleosides were confirmed with synthetic standards, while the structure of the seventh is tentatively assigned as N^4^-methyl-2′-*O*-methylcytidine (m^4^Cm) based on CID fragmentation patterns.

**Table 2.** List of modified bases detected in BCG 5S rRNA

Ribonucleoside	Abbreviation	Formula	Precursor (*m/z*)	Product (*m/z*)	Transition (*m/z*)	Retention time (min)
Adenosine	A	C_10_H_13_N_5_O_4_	268.1	136.1	132	5.09
Uracil	U	C_4_H_4_N_2_O_2_	245.1	113.1	132	2.45
Guanosine	G	C_10_H_13_N_5_O_5_	284.0	152.0	132	6.44
Cytidine	C	C_4_H_5_N_3_O	244.1	112.1	132	1.65
Inosine	I	C_10_O_5_N_4_H_12_	269.1	137.1	132	5.38
1-Methyl-adenosine	m^1^A	C_11_O_4_N_5_H_15_	282.1	150.1	132	2.53
N^6^,N^6^-Dimethyl-adenosine	m^6,6^A	C_12_O_4_N_5_H_17_	296.1	164.1	132	20.49
7-Methyl-guanosine	m^7^G	C_11_O_5_N_5_H_17_	298.1	166.1	132	4.69
1-Methyl-guanosine	m^1^G	C_11_O_5_N_5_H_15_	298.1	166.1	132	13.72
2′-O-Methyl-guanosine	Gm	C_11_O_5_N_5_H_15_	298.1	152.1	146	14.54
N^4^, 2′-*O*-Dimethyl-cytidine^a^	m^4^Cm	C_11_O_5_N_3_H_17_	272.1	112.1	160	22.64

^a^Tentative identification based on exact molecular weight and collision-induced dissociation fragmentation pattern. The identities of the other modified ribonucleosides were determined by comparisons to accurate mass measurements on an HPLC-coupled quadrupole time of flight mass spectrometer as well as retention times of chemical standards on an HPLC-coupled triple quadrupole mass spectrometer operated in positive ion mode.

## DISCUSSION

While there are established approaches for isolating specific types of RNA from mycobacteria (19–22), there are no methods that have been optimized to yield the complete set of commonly recognized ncRNA species (tRNA and 5S, 16S and 23S rRNA). The value of preserving the fidelity of a cellular RNA population by isolating the full complement of ncRNA is illustrated in recent discoveries of many new ncRNA species in *M. tuberculosis* based on genomic analyses (13,14) and with the application of quantitative RNA-seq methods to both coding and ncRNA in mycobacteria (35). Furthermore, the systems-level analysis modified ribonucleosides in mycobacterial ncRNA also requires isolation of the full spectrum of ncRNA species (15–17). To address these challenges, we used *M. bovis* BCG to develop an optimized method for mycobacterial RNA isolation that combines accessible reagents and approaches with commercial kits to achieve rapid isolation of the full spectrum of ncRNA in high yield with minimal artifacts. The method yielded high-quality ncRNA and led to the discovery of novel post-transcriptional processing of 5S rRNA in BCG.

A step-by-step analysis of the RNA isolation method provides insights into the importance of preserving both the quantitative and qualitative fidelity of the isolated RNA. Perhaps the most challenging step in mycobacterial RNA isolation involves cell lysis, owing to the unique biochemistry of mycobacterial cell walls, which renders them refractory to typical chaotropic or mild detergent-based cell lysis solutions used with other bacteria and eukaryotic cells. Several methods to overcome this problem have been developed, including enzymatic spheroplasting followed by sonication (19), detergent-based lysis (20), mechanical disruption by bead-beating (21) and mechanical shearing with a French pressure cell (22). However, in the absence of denaturants, all of these methods provide an opportunity for adventitious enzymatic reactions to occur during the lysis step, including nuclease degradation and alteration of modified ribonucleosides in the RNA. Our approach of bead-beating in the presence of a strong denaturant such as phenol:chloroform:isoamyl alcohol minimizes artifacts by reducing the time required for lysis and by quenching all physiological and biochemical processes.

It is also important to consider the choice of denaturing extraction solvents as a determinant the fidelity of the RNA population. The introduction of the ‘single-step’ method of RNA isolation using acid guanidinium thiocyanate-phenol-chloroform (i.e. TRIzol) provided versatility and ease in retrieving RNA from a wide variety of sources (36). However, Kim *et al*. recently described poor recovery of small RNA with low GC content when utilizing the isopropanol precipitation step in the TRIzol approach, challenging the assumption that total RNA preparations recover all RNA species with equal efficiency (23). We also observed that TRIzol yields a smaller proportion of 16S and 23S rRNA from mycobacteria compared to the phenol, chloroform, isoamyl alcohol mixture (Supplementary Figures S2B and S4). The alternative use of cetyltrimethylammonium bromide with phenol and bead-beating for RNA extraction from mycobacteria by Cheung *et al*. (37) proved to be problematic (20). For retrieval of RNA from the extraction solvents, an isopropanol precipitation step is frequently used, though this results in biases against small RNAs. Given these issues of extraction and recovery, we found that the combination of phenol:chloroform:isoamyl alcohol extraction with bead-beating followed by solid-phase extraction columns for RNA retrieval provided optimal RNA yields in terms of quantity and quality. As the use of phenol:chloroform:isoamyl alcohol at an alkaline pH increases the co-purification of genomic DNA, a DNA removal step, such as addition of DNase I or HPLC purification, should be employed for downstream applications involving PCR amplification or chemical analysis of RNA. The solid phase extraction technology is based on nucleic acid binding to silica-based polymers with RNA retention dependent on the concentration of salt and ethanol in the aqueous phase (38). Lower percentages of ethanol (e.g. 35%) allow retention of larger RNA while higher percentages of ethanol (e.g. 70%) retain small RNA. While there is significant loss of small RNAs, most notably tRNA, on the column supposedly retaining large RNAs (column #1 in the current method), quantitative recovery of all RNA species is achieved by combining the eluates from both columns.

This optimized approach of using denaturing extraction solvents during bead-beating to lyse mycobacteria followed by solid-phase recovery of RNA allows for rapid, efficient isolation of the complete set of ncRNA species with minimal degradation artifacts. The efficiency of the method is evident in the yield of DNA-free RNA, which amounted to a reproducible 2.6 μg of RNA from 10^9^ bacilli (i.e. 2.6 fg per cell). This amount of cellular RNA is consistent with recent studies on the molecular composition of mycobacteria that gave a theoretical total content of RNA in a single BCG bacillus at 2.2–10 fg with dependence on the growth rate (29). Given our isolation results and the agreement with theoretical RNA amounts, previous claims of 20 fg RNA per cell for mycobacteria are likely over-estimates possibly resulting from DNA or other contamination (20,37). For the highest degree of purity needed for chemical analysis of RNA, for example, individual RNA species can be purified by one-, two- or even three-dimensional chromatography combining size-exclusion with reversed-phase matrices (26). This step provides an opportunity to remove contaminating DNA and DNase I. In our experiments, we show essentially complete digestion of DNA by DNase I in order to minimize DNA contamination in purification steps (Supplementary Figure S5).

The immediate application of this method to the *M. tuberculosis* surrogate, *M. bovis* BCG, which is also the tuberculosis vaccine strain, led to three important observations: pathophysiological shifts in the relative quantities of ncRNA species, and novel post-transcriptional processing of the elusive 5S rRNA in terms of sequence trimming and an expanded set of modified ribonucleosides. Among a variety of environmental changes that occur in the hallmark pathology of *M. tuberculosis* infection, oxygen depletion *in vitro* and granulomas have been shown to induce a non-replicative persistent state that mimics the dormant state of tuberculosis bacilli (39); this phenotype is shared by BCG and the faster growing *M. smegmatis* (40). Consistent with the observation of a hypoxia-induced decrease in nucleic acid synthesis (41) and loss of both 50S and 30S ribosomal subunits (40), we observed significant decreases in the relative quantities of 16S and 23S at the fully developed non-replicative state at 21 days of hypoxia (Figure 2C). Surprisingly, the levels of small RNAs did not change significantly in the hypoxic state, either in terms of Bioanalyzer RNA profiles or HPLC quantification. Furthermore, the decrease in the relative quantity of 16S rRNA raises questions about the feasibility of using bacterial 16S rRNA, a housekeeping gene in bacterial gene expression studies, particularly those examining stress conditions. While possible, it is unlikely that the thicker cell wall of the hypoxic, non-replicative state could lead to less cell wall disruption during the cell lysis step and cause differential release of small and large ncRNA species.

The optimized RNA isolation method also yielded insights into the maturation and processing of 5S rRNA in mycobacteria. In 1978, using temperature-sensitive *rne Escherichia coli* mutants, several small RNA molecules were discovered, including 9S RNA that contained the 5S rRNA sequence (42). Further studies demonstrated that processing of 9S RNA by RNase E and/or RNase G yields what was thought to be a mature 5S rRNA (42,43). An RNase E/G homolog was identified from *M. tuberculosis* and was characterized as a 5′-endoribonuclease able to cleave putative 9S RNA *in vitro* as well as *in vivo* (44). This RNase E/G homolog, termed MycRne, was shown to cleave 9S RNA in proximity to, but upstream of, the annotated genomic 5S rRNA sequence, with the difference attributed to the association of ribosomal proteins. Using the RNA isolation method with HPLC purification, we were able to obtain a homogeneous population of 5S rRNA molecules for sequence analysis and chemical analysis of modified ribonucleosides. Sequencing of purified 5S rRNA uncovered two sites of truncation with respect to the BCG 5S rRNA gene, as shown in Figure 3B. Our findings strongly support the idea of a cleavage downstream from, but in proximity to, the start of the annotated genomic sequence, as well as a cleavage one nucleotide upstream from the end sequence to yield a functional 5S rRNA. To determine the cleavage site of 9S RNA, the previously mentioned group incubated PCR-amplified putative 9S sequences with a truncated form of MycRne protein that was overexpressed in *E. coli*. Additionally, their group utilized primer extension of total RNA to determine the position of the 5′ end of 5S rRNA. As the sequences differed by one or two nucleotides, they attributed the differences to the presence of ribosomal proteins (44). The uniformity of the observed 5S rRNA sequence in our studies rigorously establishes the functional form of this RNA.

In addition to sequence alterations, ncRNAs undergo maturation in the form of post-transcriptional insertion of dozens of different modified ribonucleosides, especially tRNA. To date, however, there have been very few observations of modified ribonucleosides in 5S rRNA in either eukaryotes or prokaryotes. Modified ribonucleosides in 5S rRNA have been described in archaea and to a smaller extent, eukaryotes (45,46). Although Yan *et al*. claim to have detected Am, Cm, m^3^C, m^6^A in mouse liver 5S rRNA, and while Cm, ac^4^C, ac^4^Cm have been detected in *Sulfolobus solfataricus, Pyrodictium occultum* and *Haloferax volcanii* 5S rRNA (46,47), the Albany Modification Database lists only four modifications in 5S rRNA: Cm, ac^4^C, ac^4^Cm and Y (48). Our analysis of BCG 5S rRNA thus adds seven more modified ribonucleosides to the 5S rRNA roster: m^1^A, m_6_^6^A, m^7^G, m^1^G, Gm, I and a putative m^4^Cm (Table 2). Given the importance of rRNA modifications in 16S and 23S rRNA in ribosome assembly and translational fidelity, as well as in mechanisms of resistance to aminoglycoside, macrolide and linezolid antibiotics (49–52), it is reasonable to assume that 5S rRNA modifications have potential effects with respect to bacterial growth and survival.

Given that the use of phenol:chloroform:isoamyl alcohol at an alkaline pH partitions nucleic acids in the organic phase (including mRNA), we sought to determine if our method was suitable for mRNA isolation or gene expression studies. We thus probed for the presence of seven mRNA transcripts on samples derived from our RNA isolation approach and samples derived from the conventional TRIzol RNA isolation approach. Analysis showed that the optimized phenol:chloroform:isoamyl alcohol approach resulted in a similar yield of mRNA when compared to TRIzol-based RNA isolation, while the RIN data attest to the quality of the total RNA preparation. We therefore believe that total RNA derived from our approach can be used for gene expression studies.

The results presented here demonstrate the utility of a mycobacterial RNA isolation method that maximally preserves the quantitative and qualitative fidelity of ncRNA species. While optimized for ncRNA, the method also provides high fidelity isolation of mRNA from mycobacteria.

## ACCESSION NUMBER

GenBank KC203333.

## SUPPLEMENTARY DATA

Supplementary Data are available at NAR Online.

SUPPLEMENTARY DATA
