# Supplementary material for: Mycobacterial RNA isolation optimized for non-coding RNA: high fidelity isolation of 5S rRNA from Mycobacterium bovis BCG reveals novel post-transcriptional processing and a complete spectrum of modified ribonucleosides
Source: Nucleic Acids Res. 2014 Dec 24;43(5):e32. doi: 10.1093/nar/gku1317 (PMC4357692; doi:10.1093/nar/gku1317)
Supplement: SUPPLEMENTARY DATA [file supp_43_5_e32__index.html]

Mycobacterial RNA isolation optimized for non-coding RNA: high fidelity isolation of 5S rRNA from Mycobacterium bovis BCG reveals novel post-transcriptional processing and a complete spectrum of modified ribonucleosides — SUPPLEMENTARY DATA 

# Mycobacterial RNA isolation optimized for non-coding RNA: high fidelity isolation of 5S rRNA from *Mycobacterium bovis* BCG reveals novel post-transcriptional processing and a complete spectrum of modified ribonucleosides

## SUPPLEMENTARY DATA

**Files in this Data Supplement:**

- SUPPLEMENTARY DATA
