# Supplementary material for: Mycobacterial RNA isolation optimized for non-coding RNA: high fidelity isolation of 5S rRNA from Mycobacterium bovis BCG reveals novel post-transcriptional processing and a complete spectrum of modified ribonucleosides
Source: Nucleic Acids Res. 2014 Dec 24;43(5):e32. doi: 10.1093/nar/gku1317 (PMC4357692; doi:10.1093/nar/gku1317)

**Supplementary Information for**

**Mycobacterial RNA isolation optimized for non-coding RNA: High fidelity isolation of 5S rRNA from *Mycobacterium bovis* BCG reveals novel post-transcriptional processing and a complete spectrum of modified ribonucleosides**

Fabian Hia, Yok Hian Chionh, Yan Ling Joy Pang, Michael S. DeMott, Megan E. McBee

and Peter C. Dedon

**Contents:**

**Supplementary Table 1.** Summary of the RNA optimization process**.**

**Supplementary Table 2**. RNA integrity numbers (RIN) and proportions of non-coding RNA.

**Supplementary Table 3**. qPCR Primers for *M. bovis* BCG str. Pasteur 1173P2.

**Supplementary Figure 1**. Growth of *M. bovis* BCG under hypoxia.

**Supplementary Figure 2**. Workflow for the isolation of total ncRNA from BCG.

**Supplementary Figure 3**. Standard curve for RNA quantification.

**Supplementary Figure 4**. Comparison of RNA extractions with phenol:chloroform:isoamyl alcohol and TRIzol.

**Supplementary Figure 5.** Size-exclusion HPLC analysis showing the removal of DNA from BCG total RNA after treatment with DNase I.

**Supplementary Figure 6.** Composite extracted ion chromatograms of modified ribonucleosides in hydrolyzed BCG 5S rRNA.


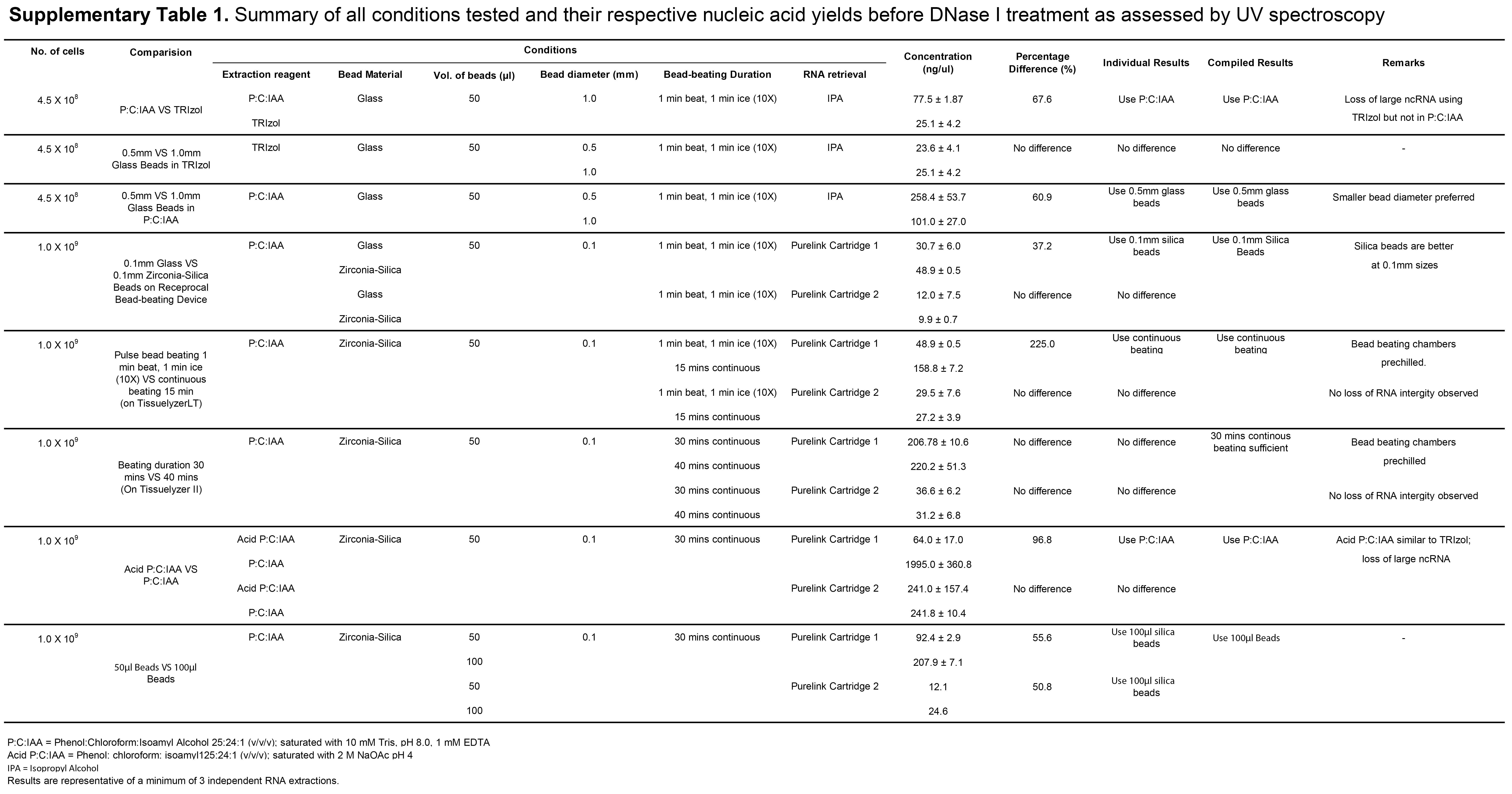


**Supplementary Table 2**. RNA integrity numbers (RIN) and proportions of non-coding RNA as determined using the Agilent RNA 6000 Pico Kit and Agilent Small RNA kit as determined by the Agilent Bioanalyzer 2100 system for **(A)** the phenol;chloroform:isoamyl alcohol approach and **(B)** the conventional TRIzol approach.

| **A** | **Bioanalyzer Chip** |  | **RNA Integrity Number** | **Percentage of RNA Eluted From Column** | | |
| --- | --- | --- | --- | --- | --- | --- |
|  |  |  |  | **23S rRNA** | **16S rRNA** | **Small RNA** |
|  | Agilent RNA 6000 Pico Kit | Column #1 | 9.0 ± 0.5 | 20.7 ± 20.7 | 17.8 ± 3.3 | 27.0 ± 3.6 |
|  |  | Column #2 | - | - | - | 95.8 ± 5.3 |
|  |  | Combined columns #1, #2 | 8.5 ± 0.3 | 16.6 ± 2.6 | 14.9 ± 1.7 | 38.9 ± 4.1 |
|  |  |  |  |  |  |  |
|  |  |  |  |  |  |  |
|  | **Bioanalyzer Chip** |  | **RNA Integrity Number** | **Percentage of RNA Eluted From Column** | | |
|  |  |  |  | **5S rRNA** | **tRNA** | **miRNA** |
|  | Agilent Small RNA kit | Column #1 | - | 17 ± 1 | 50 ± 3 | 33 ± 3 |
|  |  | Column #2 | - | 2 ± 1 | 86 ± 2 | 11 ± 1 |
|  |  | Combined columns #1, #2 | - | 12 ± 2 | 71 ± 5 | 17 ± 4 |
|  |  |  |  |  |  |  |
|  |  |  |  |  |  |  |
|  |  |  |  |  |  |  |
| **B** | **Bioanalyzer Chip** |  | **RNA Integrity Number** | **Percentage of RNA Eluted From Column** | | |
|  |  |  |  | **23S rRNA** | **16S rRNA** | **Small RNA** |
|  | Agilent RNA 6000 Pico Chip | - | - | 13.9 ± 1.4 | 10.5 ± 1.7 | 75.6 ± 3.0 |
|  |  |  |  |  |  |  |
|  |  |  |  |  |  |  |
|  | **Bioanalyzer Chip** |  | **RNA Integrity Number** | **Percentage of RNA Eluted From Column** | | |
|  |  |  |  | **5S rRNA** | **tRNA** | **miRNA** |
|  | Agilent Small RNA Chip | - | - | 8.5 ± 1.5 | 86.9 ± 2.4 | 4.6 ± 1.4 |

**Supplementary Table 3.** qPCR Primers for *M. bovis* BCG str. Pasteur 1173P2.

| **Gene** | **Direction** | **Sequence (5' - 3')** | **Melting Temperature (^o^C)** |
| --- | --- | --- | --- |
| *hspX* | F | GACGAGATGAAAGAGGGGCG | 90.0 |
|  | R | GTCGTCCTCGTCAGCACCTA |  |
| *ethA* | F | CGAGGCCGACGTTCTACTTAT | 90.0 |
|  | R | GCGACTTCGACACTGGTTGC |  |
| *fdxA* | F | AGTGAGTGCGTGGATGTGATG | 88.5 |
|  | R | TGGTGTTGATCGTCGGGTAGA |  |
| *relA* | F | ATTGCCACCAGAAACACCGA | 91.5 |
|  | R | GGTTCCGGGCGATGTGATTA |  |
| *sigA** | F | CGATGAGCCGGTAAAACGC | 91.0 |
|  | R | GAGCCACTAGCGGACTTCGC |  |
| *dosR* | F | AGTGAGTGCGTGGATGTGATG | 88.5 |
|  | R | TGGTGTTGATCGTCGGGTAGA |  |
| *dosS* | F | ATTGCCACCAGAAACACCGA | 91.5 |
|  | R | GGTTCCGGGCGATGTGATTA |  |
|  |  |  |  |
| * Primer sequence obtained from Cappelli et al. (53) | | | |

**Supplementary Figure 1.** Growth of *M. bovis* BCG under hypoxia. Cultures of BCG were sealed on day = 0 and the OD_600_ determined regularly for 21 days. The plateau in growth represents the hypoxia-induced non-replicative state.


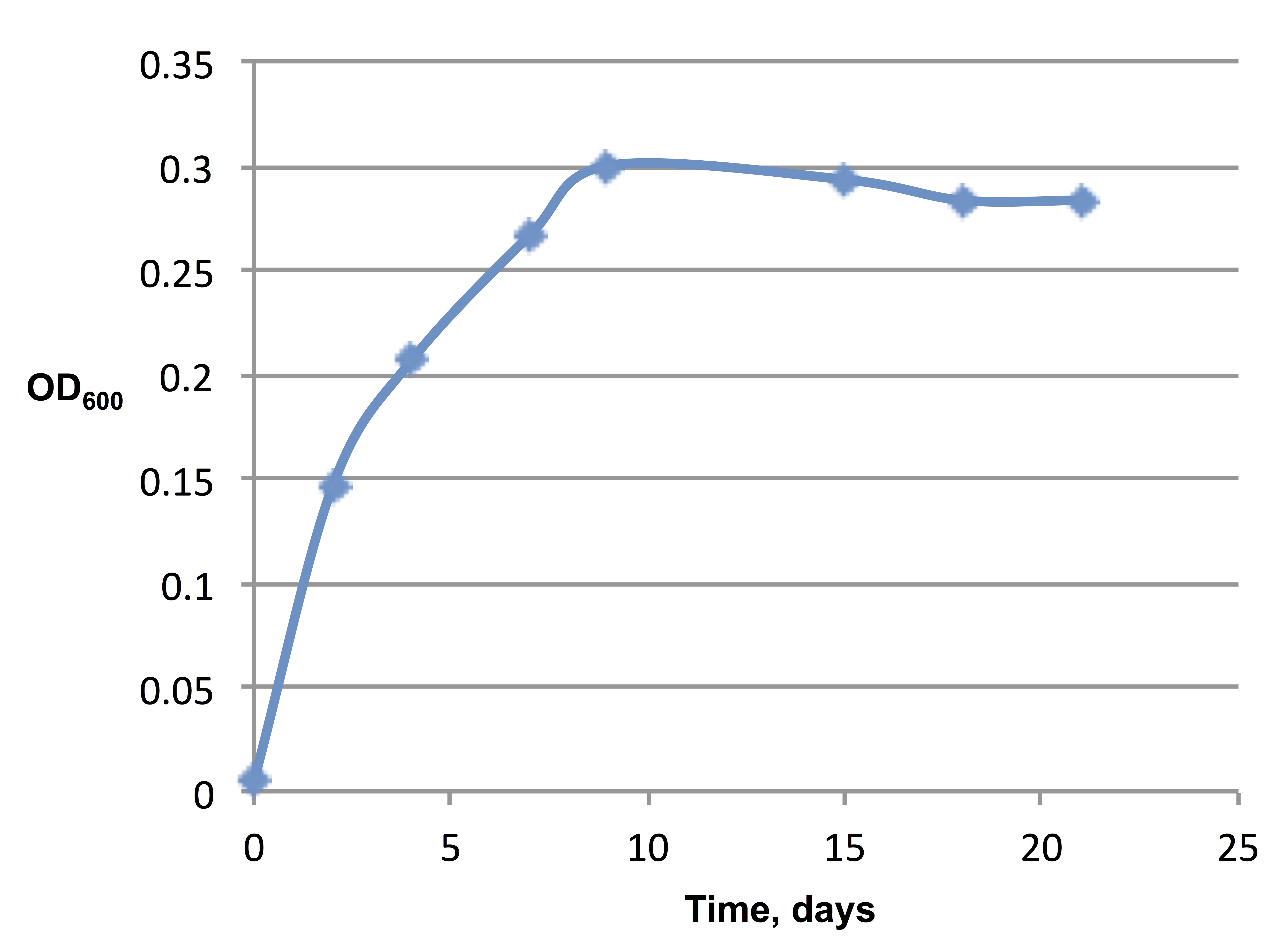


**Supplementary Figure 2**. Workflow for the isolation of total ncRNA from BCG. Bacilli are lysed by reciprocal shaking with 0.1 mm silica beads in phenol/chloroform/isoamyl alcohol and the RNA retrieved using a solid-phase extraction system (Purelink miRNA Isolation Kit). RNA from both columns is eluted and combined to yield total RNA. The ncRNA species are subsequently isolated by size-exclusion HPLC.

**Supplementary Figure 3**. Standard curve (R^2^ = 0.999) for RNA quantification as determined by HPLC analysis of 28S rRNA standards of varying concentrations.

**
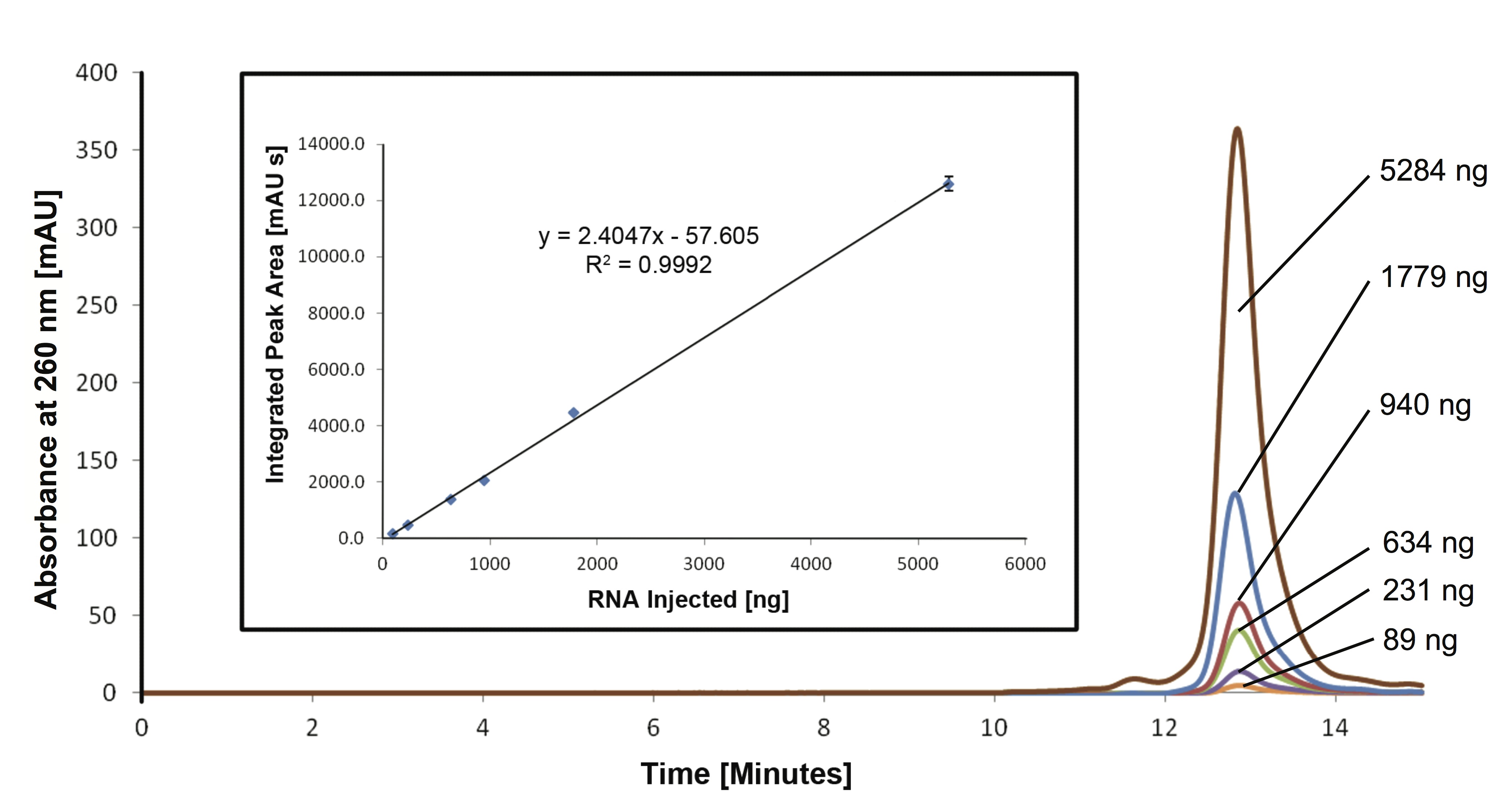
**

**Supplementary Figure 4**. Comparison of RNA extractions with phenol:chloroform:isoamyl alcohol 25:24:1 (P:C:IAA), saturated with 10 mM Tris, pH 8.0, 1 mM EDTA (Black) and TRIzol (grey). When used on BCG, significant less 16S and 23S rRNA are extracted with TRIzol when compared to phenol:chloroform:isoamyl alcohol.


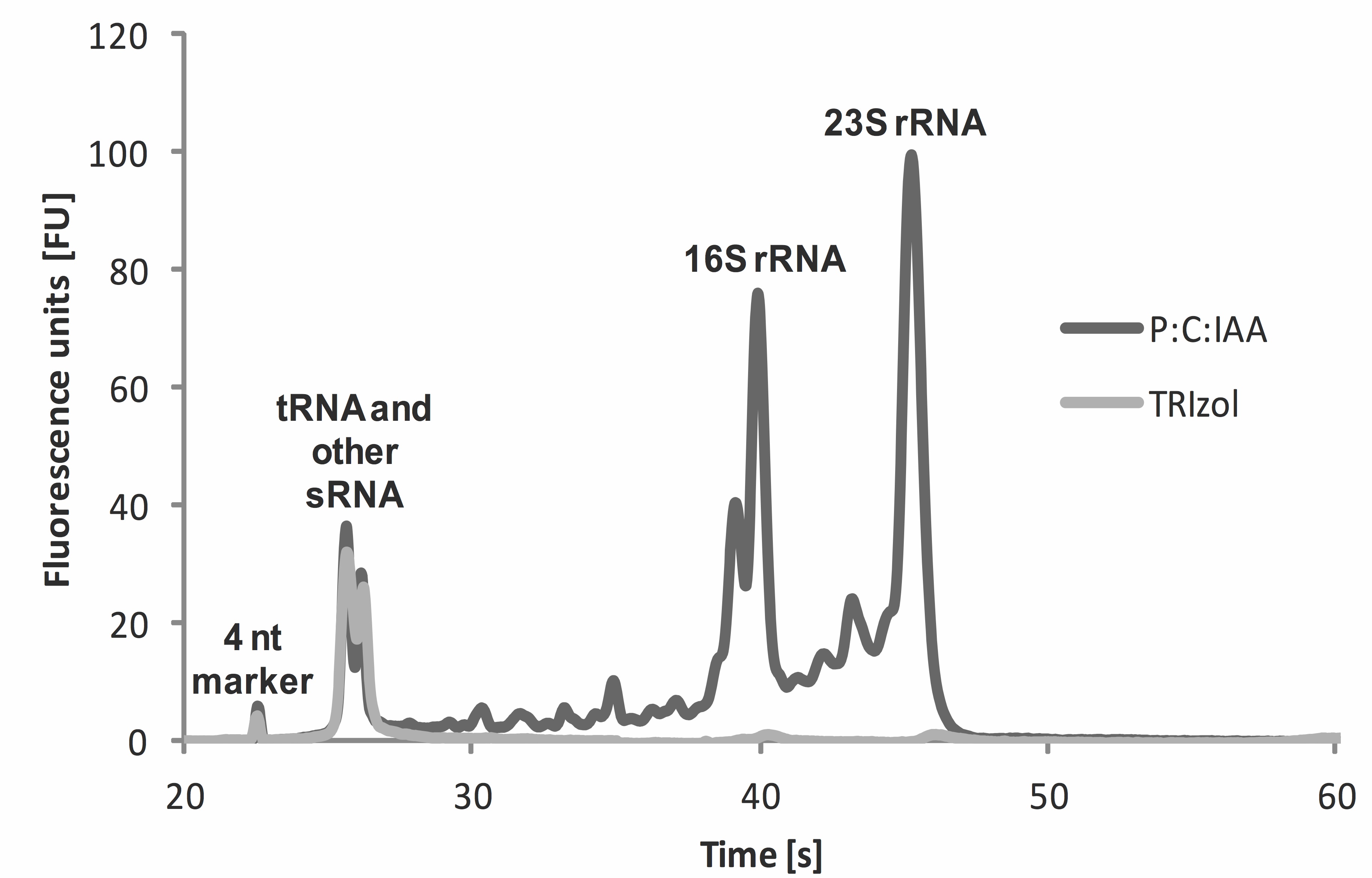


**Supplementary Figure 5.** Size-exclusion HPLC analysis showing the removal of DNA from BCG total RNA after treatment with DNase I (SEC5 1000Å column).


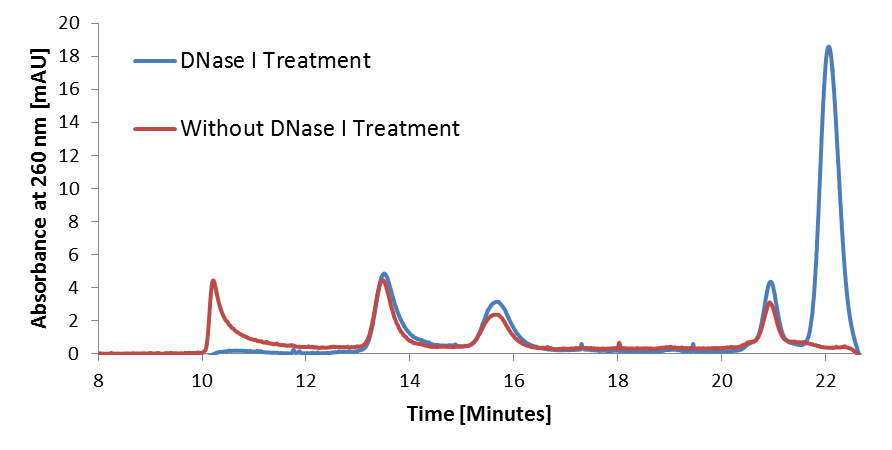


DNA

Digested

DNA

fragments

**Supplementary Figure 6.** Composite extracted ion chromatograms of modified ribonucleosides in hydrolyzed BCG 5S rRNA.


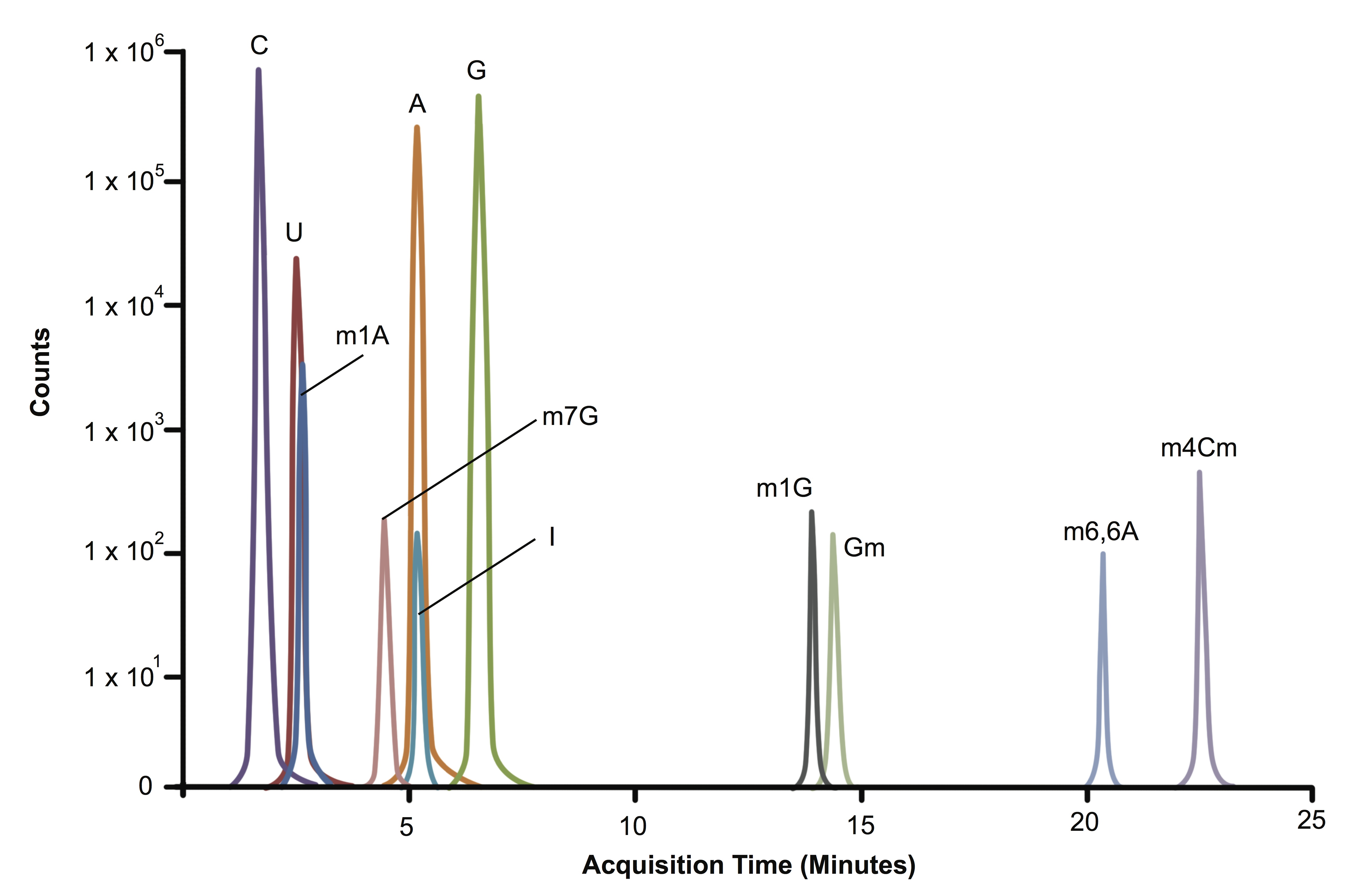

Supplement: SUPPLEMENTARY DATA [file supp_gku1317_nar-02827-met-g-2014-File005.doc]
